# Warm Water and Cool Nests Are Best. How Global Warming Might Influence Hatchling Green Turtle Swimming Performance

**DOI:** 10.1371/journal.pone.0023162

**Published:** 2011-08-03

**Authors:** David T. Booth, Andrew Evans

**Affiliations:** School of Biological Sciences, The University of Queensland, Brisbane, Australia; Institute of Marine Research, Norway

## Abstract

For sea turtles nesting on beaches surrounded by coral reefs, the most important element of hatchling recruitment is escaping predation by fish as they swim across the fringing reef, and as a consequence hatchlings that minimize their exposure to fish predation by minimizing the time spent crossing the fringing reef have a greater chance of surviving the reef crossing. One way to decrease the time required to cross the fringing reef is to maximize swimming speed. We found that both water temperature and nest temperature influence swimming performance of hatchling green turtles, but in opposite directions. Warm water increases swimming ability, with hatchling turtles swimming in warm water having a faster stroke rate, while an increase in nest temperature decreases swimming ability with hatchlings from warm nests producing less thrust per stroke.

## Introduction

All sea turtle species except the flatback turtle (*Natator depresses*) and Olive ridley turtle (*Lepidochelys olivacea*) are listed on the IUCN red list of threatened species as either endangered or critically endangered with the majority of populations continuing to decline [1]. One aspect of research directed towards sea turtle conservation has focused on identifying the impacts of impending temperature rise associated with climate change on sea turtle biology, such as elevated sea level [2], [3] and increased sand temperatures with its resultant effects on hatching success and sex ratios [4]–[11]. At rookeries surrounded by coral reefs, the biggest impediment to recruiting hatchlings into the open ocean is the high rate of predation of hatchlings by fish (which averages about 30%) as hatchling spent their first few minutes of sea life swimming across the fringing reef surrounding rookeries [12]–[14]. Along the east coast of Australia green turtles *Chelonia mydas* nest almost exclusively on islands surrounded by coral reefs [15] and thus their hatchlings must swim the gauntlet of fish predators before reaching the relative safety of the open ocean. Green turtle hatchlings appear to show no predator avoidance behavior while swimming [12] so their chance of being predated while swimming across a fringing reef is a direct function of how long it takes them to swim across the reef [12] which in turn depends to a large degree on the hatchling's swimming speed.

Temperature plays a vital role in sea turtle reproductive biology so predicted increases in temperatures will have a significant impact on their biology and conservation. All sea turtles exhibit temperature dependent sex determination where incubation at low temperatures (typically<28°C) results in males, incubation at high temperatures (typically>30°C) results in females, and incubation at intermediate temperatures results in a mixture of males and females [16]–[18]. Hence an obvious threat of global warming on sea turtle rookeries is feminization of hatchlings and potentially the complete elimination of male recruitment [4], [7], [9]–[11]. However, temperature can also influence hatchling sea turtle recruitment in more subtle ways.

Hatchling sea turtles are ectotherms, and as such their body temperature is determined by the temperature of the water they swim in, and body temperature directly affects the locomotor performance of ectotherms with locomotor performance increasing with temperature until a sub-lethal temperature is reached [19], [20]. Hence an increase in sea water temperature is expected to increase hatchling sea turtle swimming speed and thus might enhance recruitment from rookeries surrounded by coral reefs. Incubation temperature can also influences the swimming performance of sea turtle hatchlings [21] and size of sea turtle hatchlings [22], and larger green turtle hatchlings have a greater chance of surviving the crossing of the near-shore reef [13]. For these reasons we predict that an increase in incubation temperature may also change sea turtle recruitment from rookeries through its influence on hatchling size and swimming speed. In this study we examine the effects of nest and water temperature on the swimming performance of green turtle hatchlings in order to test the hypothesis that an increase in global temperature may change the recruitment of hatchling sea turtles from rookeries surrounded by coral reefs through its influence on hatchling swimming performance.

## Materials and Methods

This study was conducted on Heron Island (23°26′S, 151°51′E), a vegetated coral cay in the Capricorn-Bunker island group at the southern end of the Great Barrier Reef, Australia. Twenty ovipositing green turtles *Chelonia mydas* were located 4–10 December 2007 and their eggs collected and taken to a research station where the eggs were counted to determine clutch size and a sample of 25 eggs weighed (±0.1 g). The entire clutch of eggs was then reburied in previously dug 65 cm deep nests on the periphery of nesting area, because in a previous study a high proportion of the natural nests were destroyed by subsequent nesting females [22]. A temperature data logger (iButtons, Dallas semiconductor, Dallas USA) programmed to log temperature every 2 h was placed in the centre of the egg mass and then sand placed over the top of eggs. The nests were located along the entire length of both the north-facing and the south-facing beaches. Some nests were placed in the open and others in areas that were shaded for some part of the day in order to generate the range of nest temperatures typically experienced within a nesting season on Heron Island.

Hatchlings emerged from nests between 28 January and 16 February 2008. Nests were enclosed by 1 m diameter plastic mesh (18 cm high) carrels in the late afternoon and visited even two hours throughout the night. Upon emergence from a nest a sample of 25 hatchlings was taken back to the laboratory and each hatchling weighed (±0.1 g) and straight carapace length and carapace width at its widest point measured (±0.1 mm) with a digital caliper. A size index was calculated by multiplying carapace length by carapace width. A sample of eight hatchlings from each clutch had their swimming performance measured in a four-hour swimming trial following the method of Ischer et al. [22] four hatchlings being swum at 26°C and the other four being swum at 30°C as these are the typical minimum and maximum water temperatures regularly experienced on the fringing reef at Heron Island during the hatching season (January–March) [23]. This method involves tethering hatchlings to a force transducer and sampling the thrust (force) generated 40 times per second. Two days after the first hatchlings emerged, nests were excavated to retrieve temperature data loggers. Mean nest temperature (here after termed nest temperature) was calculated by averaging all temperature measurements from the time eggs were buried until hatching which was determined by a sudden decrease in nest temperature [6].

**Figure 1:**
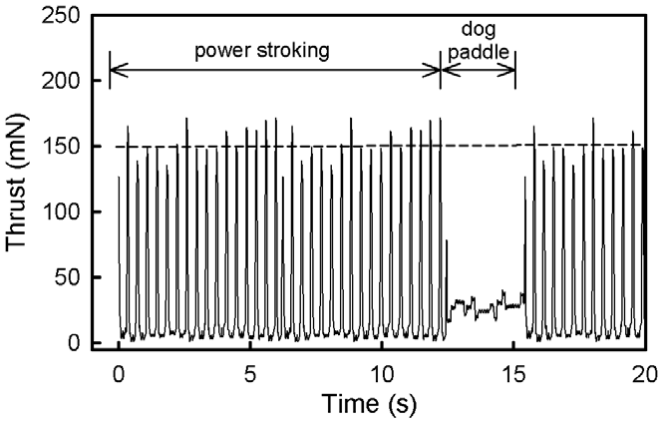
A twenty second snapshot of a typically green turtle hatchling thrust trace while swimming showing power-stroking and dogpaddling bouts. Dashed line indicates the mean peak thrust for the power-stroking bouts.

Green turtle hatchlings engage in a highly stereotypical swimming behavior as soon as they enter the water where 5–15 s bouts of power-stroking which involving the up and down movement of the front flippers are separated by 3–10 s bouts of dogpaddling where the head is lifted above the water and a breath of air taken while the front left and rear right and front right and rear left flippers are moved in unison [22], [24], [25] (Fig. 1). The four hour swimming trial was divided into 10 minute periods so that temporal changes in swimming performance could be followed across the entire swimming trial. Swimming performance was assessed by calculating four variables. Mean thrust was calculated by averaging all 24000 sampling points in a ten minute sampling period. This is the best overall measure of swimming performance because it is determined by the combined effects of peak thrust of power-strokes, the power-stroking rate and the proportion of time spent power-stroking. The relative change in these three variables with time explain why mean thrust changes with time. Peak thrust was calculated by examining thrust traces and estimating the mean peak thrust generated during each power-stroke for each 10 minute period (Fig. 1). Power-stroke frequency was calculated from the power-stroke frequency during a power-stroking bout during each ten minute period. Percent time spent power-stroking was calculated by counting the actual number of power strokes taken in each 10 minute period (which included power stroking bouts and dog paddling bouts) and dividing this by 10×(the power stroke rate during a power stroking bout for that ten minute period) and converting this to a percentage by multiplying by 100.

Correlation and least squares regression were used to investigate relationships between egg mass, hatchling mass and size index, and between nest temperature and hatchling mass and size index. ANOVA with clutch as a random factor was used to compare egg mass between clutches. Because initial egg mass varied significantly between different clutches, and initial egg mass directly influences the hatchling mass and size index, ANCOVA with egg mass as the covariate and nest as a random factor was used to compare hatchling mass and size between nests. This statistical procedure essentially adjusts hatchling mass and size for differences in initial egg mass. ANCOVA generated least squares means for each nest (i.e. hatchling mass and size adjusted for inter-clutch differences in initial egg mass) were used in correlation analysis between nest temperature and hatchling mass and size. Repeat measures ANOVA with water temperature and incubation environment (warm/cool) as fixed factors were used to compare hatchling swimming characteristics. Statistica release 8 was used for all statistical analysis and statistical significance assumed if P<0.05.

## Results

### Nest temperature and body size

One nest was destroyed during incubation, but the other 19 nests hatched successfully and were used to obtain hatchling mass and size data. However, only hatchlings from 12 nests were swum (Table 1) because the other seven nests hatched while the swimming setup was being used by hatchlings that emerged earlier that night.

**Table 1:** Mean initial egg mass, nest temperature and hatchling mass and size index of the 19 successfully hatched nests.

Nest number	Egg mass (g)	Nest temperature (°C)	Nest warm or cool?	Hatchling mass (g)	Hatchling size index (mm^2^)	Sample of hatchlings swum?
1	54.6±0.4	29.3	Cool	25.8±0.2	1913±19	Yes
2	54.5±0.4	29.5	Cool	25.0±0.3	1848±22	Yes
4	49.3±0.4	30.3	Warm	23.7±0.2	1757±19	Yes
5	55.9±0.4	29.6	Cool	27.3±0.2	1890±18	Yes
6	55.7±0.4	28.2		27.6±0.2	2040±20	No
7	53.9±0.4	29.6	Cool	27.2±0.2	1818±18	Yes
8	54.2±0.4	28.6		26.0±0.2	2110±18	No
9	56.0±0.4	28.4		26.6±0.2	1866±20	No
10	56.5±0.4	28.4		28.5±0.2	1885±18	No
12	45.3±0.4	29.2	Cool	22.3±0.2	1825±18	Yes
13	49.1±0.4	30.7	Warm	25.3±0.2	1705±18	Yes
14	59.6±0.4	32.3	Warm	25.6±0.2	1823±19	Yes
15	60.0±0.4	28.7	Cool	28.6±0.2	2041±18	Yes
16	50.6±0.4	29.3		24.2±0.2	1849±18	No
17	52.5±0.4	29.3		25.7±0.2	1872±18	No
18	50.4±0.4	27.6	Cool	25.4±0.2	1796±18	Yes
19	60.1±0.4	30.1	Warm	27.6±0.2	1873±18	Yes
21	48.8±0.4	30.2	Warm	24.8±0.2	1789±18	Yes
22	48.3±0.4	29.2		25.1±0.2	1868±18	No
Warm nest mean	53.8±1.7	30.7±0.4		25.4±0.7	1788±28	
Cool nest mean	53.5±2.7	29.1±0.3		26.0±0.2	1899±32	

N = 25 for both eggs and hatchlings from each nest. Nests that had a sample of hatchlings swum were classified as either warm (>30°C) or cool (<30°C).

**Figure 2:**
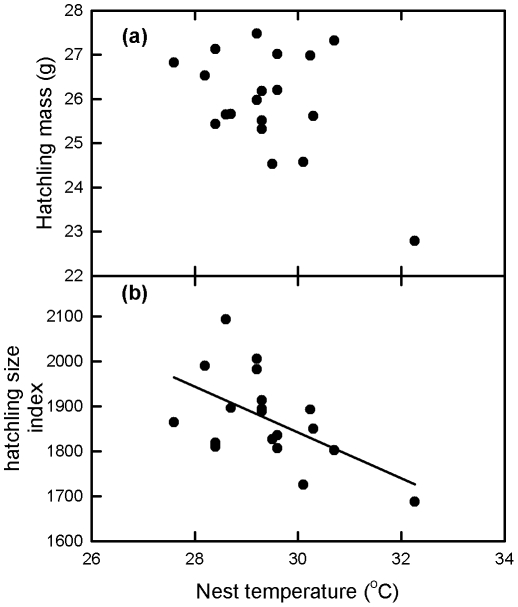
Scatter plots of ANCOVA (egg mass covariate) least square mean green turtle hatching mass and hatchling size index against nest temperature. (A). Hatchling mass. No significant correlation (r^2^ = 0.22, P = 0.058, N = 19). (B). Hatchling size index. (size index = 3367−50.84×nest temperature, r^2^ = 0.30, P = 0.015, N = 19).

Egg and hatchling mass and hatchling size index varied significantly between nests (Table 1, P<0.001 in all cases), and these inter-nest differences in hatchling mass and size index persisted even after egg mass was included as a covariate (P<0.001 in both cases). Hatchling mass and hatchling size index were correlated with egg mass (hatchling mass = 0.3344×egg mass+7.664, r^2^ = 0.62, P<0.001, N = 475; hatchling size index = 13.79×egg mass+1139, r^2^ = 0.22, P<0.001, N = 475). Nest temperature varied between 27.6°C and 32.3°C (Table 1). There was no significant correlation between nest temperature and hatchling mass, but there was a significant negative correlation between nest temperature and hatchling size index (Fig. 2).

### Nest temperature, water temperature and swimming performance

**Figure 3:**
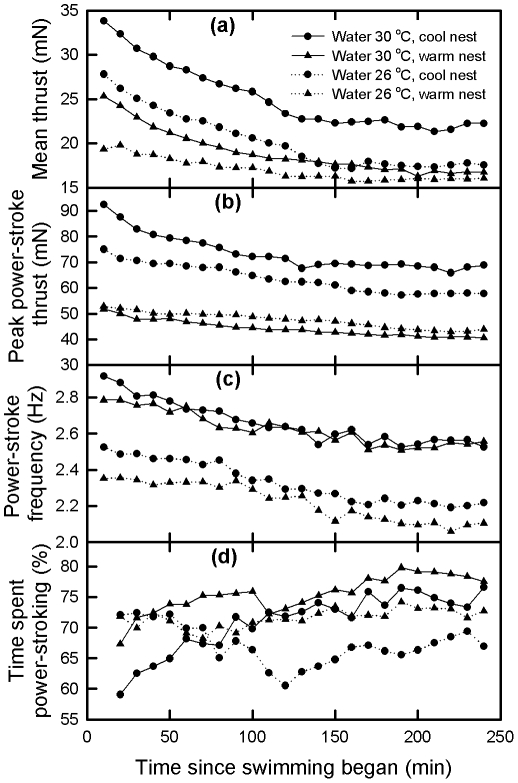
Plots of (a) Mean thrust, (b) Peak power-stroke thrust, (b) Power-stroke frequency, and (d) Percent of time spent power stroking of green turtle hatchlings during a four hour swimming trial and swum in water at either 26°C or 30°C. A total of 28 hatchlings from cool nests (4 from each of 7 nests) were swum at each of the two water temperatures (i.e. 28 swum at 26°C and another 28 swum at 30°C). A total of 20 hatchlings from warm nests (4 from each of 5 nests) were swum at each of the two water temperatures (i.e. 20 swum at 26°C and another 20 swum at 30°C).

**Figure 4:**
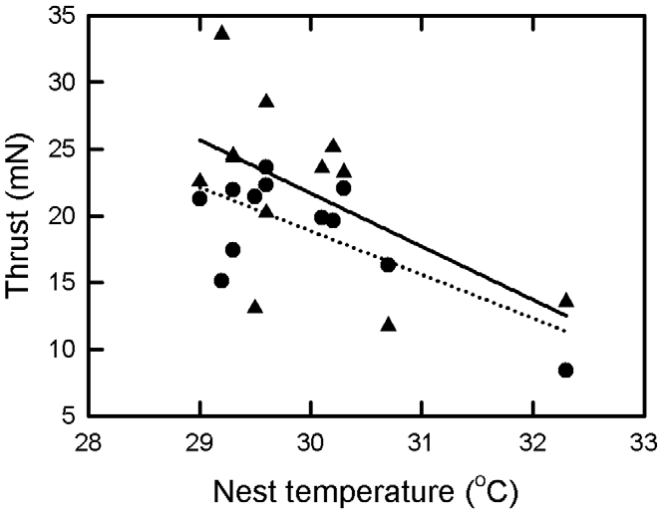
Scatter plots of green turtle hatchling mean thrust over a four hour swimming trial against nest temperature at two water temperatures. Circles and dotted line represent hatchlings swum at 26°C (thrust = 117.2−3.28×nest temperature, r^2^ = 0.49, P = 0.012, N = 12 nests). Triangles and solid line represent hatchlings swum at 30°C (thrust = 141.7−4.00×nest temperature, r^2^ = 0.31, P = 0.049, N = 12 nest). Nest values are the means of the four hatchlings from each nest swum at each temperature.

To discover if nest temperature influenced swimming performance, nests that had hatchlings swum were classified as either warm (30–32°C) or cool (27–30°C) (Table 1). After accounting for differences in egg mass, there was no difference in hatchling mass (P = 0.488), but significant differences in hatchling size index (P = 0.012) between warm and cool nests, cool nests producing hatchlings with a larger size index. Repeat measures ANOVA with time during the swimming trial the repeated factor, and nest temperature (warm or cool) and water temperature (26°C or 30°C) as fixed factors indicated that mean thrust (P<0.001), peak thrust (P<0.001) and power-stroke frequency during a power stroke bout (P<0.001) decreased with swimming time (Fig. 3a,b,c). There was a significant time*water temperature interaction (P<0.001) in the proportion of time hatchlings spent power-stroking such that hatchlings swum in 30°C water increased the proportion of time they spent power-stroking as swimming time increased, but hatchlings swum in 26°C water did not change the proportion of time they spent power-stroking as swimming time increased (Fig. 3d). There was no affect of nest temperature (P = 0.159) on proportion of time spent power-stroking. Both water temperature (P = 0.041) and nest temperature (P = 0.006) influenced mean thrust, with hatchlings from cool nests swum in 30°C water being the strongest swimmers, followed by hatchlings from cool nests swum at 26°C, then hatchlings from warm nests swum at 30°C, and hatchlings from warm nests swum at 26°C being the weakest swimmers (Fig. 3a). However there were significant water temperature*nest temperature (P = 0.026), time*water temperature (P<0.001) and time*nest temperature (P<0.001) interactions so that after 120 minutes of swimming, swim thrust of hatchlings remained the greatest in hatchlings from cool nests swum in water at 30°C, but swim thrust of hatchlings from the other nest temperature/water temperature combinations were similar to each other (Fig. 3a). When swim thrust was averaged across the entire four hour swimming trial, both water (P<0.001) and nest temperature (P<0.001) were found to influence swimming performance, with hatchlings swimming in 30°C water generating greater thrust than hatchlings swimming in 26°C water, and hatchlings from cool nests generating greater thrust than hatchlings from warm nests. Swim thrust was correlated with nest temperature in hatchlings swum in water at 26°C and 30°C (Fig. 4). Nest temperature (P = 0.003) but not water temperature (P = 0.654) was found to influence peak thrust, with hatchlings from cool nests producing greater peak thrust (Fig. 3b). Water temperature (P<0.001) but not nest temperature (P = 0.141) was found to influence power stroke frequency during a power-stroking bout, with hatchlings swum in 30°C water having a greater stroke frequency (Fig. 3c). Despite the fact that there was only a relative small range in body size of hatchlings, there was a weak positive correlation between thrust averaged over the four hour swimming trial and hatchling size index (mean thrust = 0.0175×size index−11.6, r^2^ = 0.06, P = 0.022, N = 96).

## Discussion

### Nest temperature now and into the future

Every sea turtle nest as a unique temperature profile, but different temperature profiles can have the same nest temperature when the temperature profile is averaged throughout the incubation period. Factors known to influence the nest temperature profile are sand color, beach orientation, amount of direct sunshine or shade a nest receives, nest depth which interacts with whether the beach sand temperature is rising during the beginning of the nesting season, or decreasing towards the end of the nesting season, and the amount of metabolic heat generated by the developing embryos (which in turn depends on the size and number of viable eggs in a clutch) [26], [27]. In the current study nests we located on the north and south facing beaches, and placed in full sun or various degrees of shade in order to generate the wide range of nest temperatures (4.7°C, Table 1) which might be encountered on Heron Island in a typical nesting season. Global air temperatures and those in the Great Barrier Reef are predicted to rise between 1.9°C and 2.6°C by 2050 [28], [29] and sea surface temperature to rise by 1.6°C [29]. Sea surface temperature during January–March the peak hatching season currently averages ∼28°C [23] so it is expected to reach ∼30°C (the upper water temperature used in the swimming performance tests in the current study) by 2050. Seasonal increases and decreases in sand temperature at nest depth at Heron Island parallel seasonal changes in air temperature [26], [27] so it is expected that a 2°C rise in air temperature will result in a 2°C rise in sand temperature at nest depth, and that as a result mean nest temperature will rise from 29.5°C (now) to 31.5°C (2050). The range of variation in nest temperature is not expected to change with an increase in overall climatic temperature and to be about the same as it currently is ∼5°C for Heron Island (Table 1).

### Nest temperature and body size

Both clutch of origin and nest temperature influenced body size of green turtle hatchlings. Clearly hatchling mass and size are highly dependent on egg mass, and inter-nest differences in egg mass can explain some of the difference between hatchling mass and size. However, even when egg mass is factored in as a covariate, inter-nest differences in hatchling mass and size index persisted indicating that differences in egg mass alone cannot explain inter-nest differences in these variables. Differences in egg quality such as different proportions of yolk and albumen in an egg (eggs with proportionally larger yolks may result in larger and heavier hatchlings) may explain inter-nest differences in hatchling mass and size, along with differences in the incubation environment between nests. Nest temperature was correlated with hatchling size index, but not hatchling mass in the current study, with warm nests producing hatchlings of similar mass but smaller carapace dimensions than cool nests. This finding is consistent with the hypothesis that incubation at higher temperatures reduces the amount of yolk converted to hatchling tissue due to a shortened incubation period such that hatching from warm nests hatch with smaller carapace dimensions but a larger residual yolk than hatchling from cool nests [30], [31]. Previous studies have found the same trend in green turtle and loggerhead turtle hatchlings (see [22] for review).

We can only speculate on the ecological relevance (if any) of the relative small adjustments to body size caused by differences in nest temperature. Larger body size may be advantageous if hatchlings are entering a predator rich environment in which some predators are gape-limited and can only take smaller prey. Also larger hatchlings tended to generate greater thrust which may make them faster swimmers and decrease the time spent crossing the fringing reef and thus decrease their probability of being eaten by a predator because they are exposed to the predator-rich shallow water for a shorter time. Indeed, larger green turtle hatchlings from Heron Island have a greater probability of surviving the swim across the fringing reef than smaller individuals [13]. On the other hand, if hatchlings are entering a food-poor environment (which may be the case because hatchlings feed on zooplankton in off-shore waters [32] which has a patchy distribution), having a smaller body size but larger yolk reserve may allow them to survive starvation longer than larger bodied hatchlings which have smaller yolk reserves [22].

### Nest temperature, water temperature and swimming performance

The changes in swimming performance with time of green turtle hatchlings in this study are consistent with those reported previously for this species, i.e., a relative rapid decrease in swimming effort over the first two hours followed by a much slower decrease [22], [25]. Both peak mean power-stroke thrust and power-stroke frequency during a power-stroking also reflect this pattern. However, the proportion of time spent power-stroking either didn't change with time (hatchlings swum at 26°C) or increased slightly with time (hatchlings swum at 30°C). Both water temperature and nest temperature influenced hatchling swimming performance as measured by mean thrust, with hatchlings from cool nests swum at 30°C being the most vigorous swimmers, and hatchlings from warm nests swum at 26°C being the least vigorous swimmers. Nest temperature was correlated with thrust of hatchlings swum at 26°C and at 30°C indicating that nest temperature plays a role in determining hatchling swimming performance independent of water temperature. Examining the different components (peak thrust, power-stroke frequency, and percent time spent power-stroking) that contribute to mean thrust revealed that nest temperature and water temperature affect these components in different ways.

Nest temperature's main affect on overall thrust production was through its effect on the peak power-stroke thrust produced, hatchlings from cooler nests generated greater peak thrust than hatchlings from warmer nest. Water temperature had no significant effect on peak thrust generated by hatchlings. A possible explanation for the difference in peak thrust produced by hatchling from warm and cool nest is that during embryonic development nest temperature influences the development of muscle fibers within front limb muscles that drive the power-stroking process. Incubation temperature during development of fish embryos has been demonstrated to influence the number and type of muscle fibers in larval fish [33] so a similar process could occur in sea turtle embryos. This hypothesis could be tested by examining the physiological and mechanical properties of isolated muscle preparations sampled from forelimb muscles of hatchling turtles that developed at different temperatures and by histological examination of fiber type within these muscles.

The influence of water temperature on overall swimming performance was evident through its effect on power-stroke frequency during a power-stroking bout; hatchlings swum in 30°C water had stroking frequencies greater than those swum in 26°C water. Nest temperature had no significant effect on stroking frequency. The most likely explanation for this observation is a general increase in muscle metabolism and general increase in delivery capacity by the cardiovascular system with an increase in body temperature. An increase in heart rate with temperature is a common phenomenon in ectothermic vertebrates [34] and can result in an increase in the rate of delivery of nutrients and oxygen and an increase in the rate of removal of metabolic waste products and carbon dioxide from muscles and thus facilitate an increase in muscle activity. An increase in muscle temperature will also increase the rate of biochemical reactions within muscles and thus increase muscle metabolism and performance [34]. A combination of these two effects probably explains the increase in power-stroke frequency observed at higher water temperature, because turtle hatchlings are ectotherms and their body temperature closely approximates water temperature.

### Ecological implications

For hatchling sea turtles that emerge from nests on beaches surrounded by coral reefs, one of the highest causes of mortality is fish predation as hatchlings swim across the predator rich fringing reef while making their way to the relative safety of deeper oceanic waters [12]–[14], [35]. At Heron Island the majority of green turtle hatchlings swim in a direct line from the beach to the reef crest [12] and swim distances between 100 m and 1000 m to cross the fringing reef [12], so the frantic swim from the shore to deeper water takes a relative short time (typically 15–60 minutes depending on the width of the fringing reef [12], [14]). Green turtle hatchlings cannot swim fast enough to out-swim or out maneuver their fish predators, and do not appear to try and avoid predation, their only anti-predator strategy is to spend as little time as possible in the shallow near-shore fringe reef waters [12]. Predation rate of hatchlings is not constant and is known to vary with the tidal cycle (higher predation rate on the low tide when the water is shallower), and with moon phase (higher predation when the moon is full or in the first and third quarters) [12]. However, hatchlings do not time their entry into the sea to coincide with times of minimal predation pressure; they enter the sea soon after emerging from their nest no matter what the tide or moon conditions. Hence for any particular combination of tidal, moon or predation conditions, the chance of predation should be proportional to the time spent swimming across the reef. Under such circumstances the faster a hatchling can swim, the greater its chance of making it to the relative safety of deeper off-shore water. For this reason, even small changes in a hatchling's swimming speed can significantly affect its chance of survival. Although swimming speed was not measured directly in the current study, hatchlings were of similar size and shape and therefore had a similar hydrodynamic resistance to movement through water. In such circumstances greater thrust production results in increased speed, so mean thrust can be considered a reasonable proxy for swim speed [22], [25]. We found that in the 2007–2008 nesting season higher nest temperature and lower water temperature resulted in lower swim thrust production and by implication slower swim speed and a higher probability of being predated by a fish while crossing the fringing reef.

The current study indicates that global warming may affect hatchling sea turtle swimming ability and hence survival through its effects on increasing nest and sea surface temperatures. Interestingly the effects are in opposite directions. An increase in sea surface temperature is predicted to result in an increased swimming speed, while an increase in nest temperature is predicted to result in a decreased swimming speed. However, based on the data gathered in the current study, we predict that the negative effect on swimming caused by an increase in nest temperature will be greater than the positive effect of an increase in water temperature so that the overall effect will probably be a decrease in swimming ability which may result in decreased hatchling recruitment. Further research aimed at measuring actual recruitment rates of sea turtles under different thermal regimes are needed to test these predictions.
